# No benefit of the combination therapy etanercept and methotrexate compared to etanercept mono therapy in juvenile idiopathic arthritis – a matched pair analysis

**DOI:** 10.1186/1546-0096-10-S1-A59

**Published:** 2012-07-13

**Authors:** Heinrike Schmeling, Gerd Horneff

**Affiliations:** 1 Asklepios Clinics, Sankt Augustin, Nordrhein-Westfalen, Germany; 2 University of Calgary, Calgary, AL, Canada

## Purpose

Etanercept has been approved in polyarticular juvenile idiopathic arthritis (JIA) patients refractory or intolerant to methotrexate. The objectives of the study were to evaluate safety and efficacy of Etanercept and Methotrexate combination therapy versus Etanercept mono therapy in JIA using a matched pair analysis.

## Methods

A cohort of 1190 JIA patients enrolled in the German JIA Enbrel registry has been used for screening. Matching criteria included JIA subtype, gender, disease duration, use of corticosteroids, oligo- versus polyarthritis at therapy start and the sedimentation rate. Efficacy was determined using the PedACR 30/ 50 and 70 response criteria. Safety assessments were based on adverse events reports. The matched data at month 1, 3, 6, 12, 18 and 24 on treatment have been analyzed using the McNemar’s test.

## Results

87 JIA matching pairs with a total of 658 visits have been identified. No difference in PedACR 30/50/70 treatment response between mono and combination therapy has been found: month 1: 73%/61%/33% vs. 70%/61%/33%, month 3: 73%/69%/51% vs. 65%/59%/41%; month 6: 82%/77%/59% vs. 83%/72%/59%; month 12: 85%/78%/63% vs. 89%/80%/67%; month 18: 77%/73%/69% vs. 89%/81%/77%; month 24: 63%/56%/50% vs. 56%/34%/34%. In the mono therapy group 18 patients (21%) experienced 28 adverse events. Five of them have been reported as serious. In the combination group 19 patients (22%) experienced 30 adverse events. Seven of them have been reported as serious. No infectious serious adverse events occurred in the mono therapy group compared to 3 in the combination group. Two malignancies have been observed in the combination group, none in the monotherapy group. Treatment has been discontinued in 29 patients on mono therapy (inefficacy 10%, adverse events 8%, remission 21%, others 16%) compared to 51 patients in the combination group (inefficacy 21%, adverse events 8%, remission 18%, others 36%).

## Conclusion

In contrast to the wide spread belief, combination treatment with Etanercept and Methotrexate did not offer a better outcome than treatment with Etanercept alone in JIA patients who previously have been treated ineffectively with methotrexate. However the rate of serious adverse events, especially serious infections was slightly increased.

## Disclosure

Heinrike Schmeling: None; Gerd Horneff: Abbott Immunology Pharmaceuticals, 2, 5, Bristol-Myers Squibb, 5, Chugai, 5, Nycomed, 5, Pfizer Inc, 2, 5, Sandoz, 5.

